# A genomic deletion encompassing *CRYBB2-CRYBB2P1* is responsible for autosomal recessive congenital cataracts

**DOI:** 10.1038/s41439-022-00208-7

**Published:** 2022-09-08

**Authors:** Bushra Irum, Firoz Kabir, Nadav Shoshany, Shahid Y. Khan, Bushra Rauf, Muhammad Asif Naeem, Tanveer A. Qaiser, Sheikh Riazuddin, J. Fielding Hejtmancik, S. Amer Riazuddin

**Affiliations:** 1 The Wilmer Eye Institute, Johns Hopkins University School of Medicine, Baltimore, MD 21287 USA; 2 National Centre of Excellence in Molecular Biology, University of the Punjab, Lahore, 53700 Pakistan; 3 Ophthalmic Genetics and Visual Function Branch, National Eye Institute, National Institutes of Health, Bethesda, MD 20892 USA; 4 Jinnah Burn & Reconstructive Surgery Centre, Jinnah Hospital, Lahore, 54550 Pakistan

**Keywords:** Disease genetics, Genetics research

## Abstract

Here we report a consanguineous Pakistani family with multiple affected individuals with autosomal recessive congenital cataract (arCC). Exclusion analysis established linkage to chromosome 22q, and Sanger sequencing coupled with PCR-based chromosome walking identified a large homozygous genomic deletion. Our data suggest that this deletion leads to *CRYBB2-CRYBB2P1* fusion, consisting of exons 1–5 of *CRYBB2* and exon 6 of *CRYBB2P1*, the latter of which harbors the c.463 C > T (p.Gln155*) mutation, and is responsible for arCC.

According to the Cat-Map database (https://cat-map.wustl.edu), 38 mutations identified in *CRYBB2* are associated with autosomal dominant cataract (adC). *CRYBB2* and its pseudogene *CRYBB2P1* reside on chromosome 22, nearly 250 kb apart^1^. The gene conversion that shifts the NM_000496.3:c.463 C > T [p.(Gln155*)] variation from the pseudogene to *CRYBB2* has been reported in different ethnic backgrounds segregating as an autosomal dominant trait (https://cat-map.wustl.edu).

A family (PKCC212) was included for a collaborative study to investigate the genetic basis of arCC. Institutional Review Board (IRB) approvals were obtained from Johns Hopkins University School of Medicine (Baltimore, MD), the National Institutes of Health (Bethesda, MD), and the National Centre of Excellence in Molecular Biology (Lahore, Pakistan). The study was completed in accordance with the Declaration of Helsinki, and all participants signed informed consent before enrollment.

Ophthalmic examinations, including slit-lamp microscopy, were performed at the Layton Rahmatulla Benevolent Trust (LRBT) Hospital (Lahore, Pakistan). Approximately 10 ml of blood was drawn from all subjects and stored in 50-ml Sterilin Falcon tubes with 20 mM EDTA. Genomic DNA was extracted, and exclusion analysis for reported arCC genes/loci using polymorphic short tandem repeat (STR) markers and Sanger sequencing were performed, as described^2,3^.

A total of eight individuals were enrolled in the study (Fig. 1a). The medical records of ophthalmic examinations confirmed that all three affected individuals had bilateral nuclear cataracts; surgery was performed on all the affected individuals except for individual V:3, before enrollment in the study (Fig. 1b). Importantly, unaffected family members exhibited no lens opacity, bilaterally. As shown in Supplementary Figs. 1 and 2, unaffected individuals IV:3 (age, 41 years) and V:1 (age, 16 years) had no signs of cataracts.

**Fig. 1:**
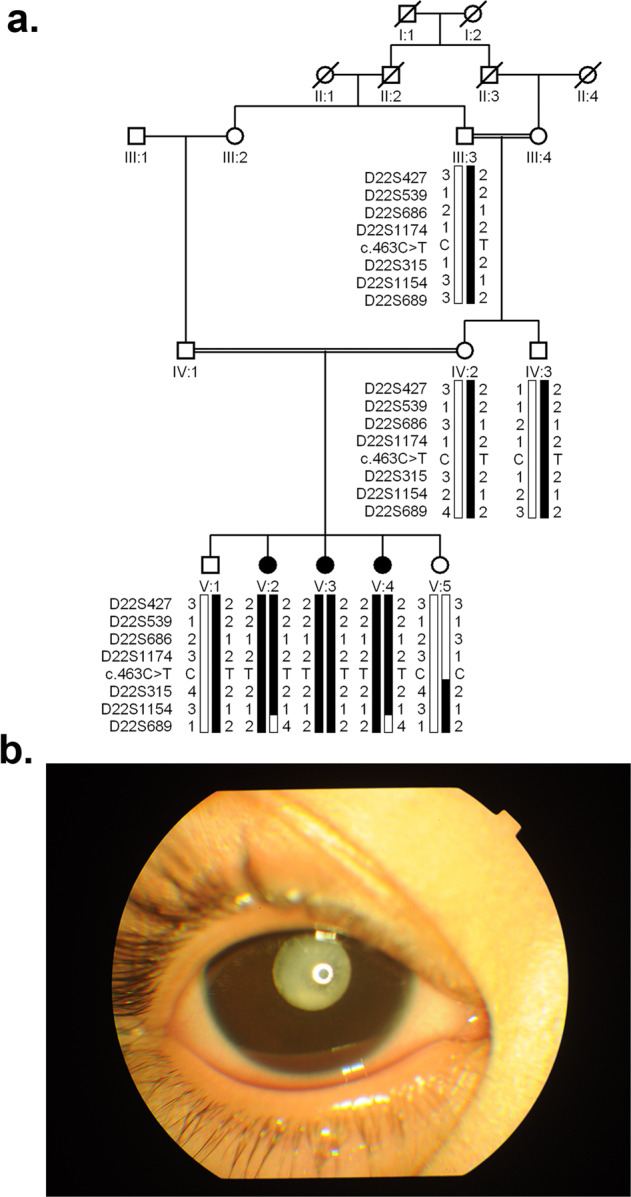
A consanguineous family, PKCC212, with three members having cataracts. **a** Pedigree drawing of PKCC212 with the haplotypes of alleles for chromosome 22q microsatellite markers. Alleles forming the risk haplotype are shaded black, and alleles not cosegregating with arCC are shown in white. Square: male; circle: female; filled symbol: affected individual; the double line between individuals: consanguineous marriage; diagonal line through a symbol: deceased. **b** Slit-lamp photograph of affected individual V:3 in the PKCC212 showing nuclear cataracts.

Exclusion analysis confirmed linkage to chromosome 22q11.23, with a maximum two-point LOD score of 2.51 for marker D22S315 (Supplementary Table 1). Sanger sequencing excluded the possibility of pathogenic variants in *CRYBA4, CRYBB1, CRYBB3*, and in the first four coding exons (2–5) of *CRYBB2*. However, a primer pair specific for exon 6 of *CRYBB2* failed to amplify, indicative of a genomic deletion and/or a rearrangement. We designed primer pairs to amplify the regions between *CRYBB2* and *LRP5L* (BB2_LRP), exon 2 of *LRP5L* (LRP5L_Ex2), intron 3 of *LRP5L* (LRP5L_Int3), and a region of *CRYBB2P1* (BB2P1_Int) (Supplementary Table 2). PCR failed to amplify all the abovementioned regions in affected individuals, but PCR products were obtained with genomic DNA from unaffected individuals i.e., III:3, IV:2, IV:3, V:1, and V:5 (Supplementary Fig. 3), suggesting a large genomic deletion.

In our effort to determine the extent of the deletion, we examined the PCR product of a forward primer annealing to *CRYBB2* and a reverse primer annealing to *CRYBB2P1* (Supplementary Table 2; BB2Int5_BB2P1Ex6), which theoretically would amplify an ~230-kb region, chr22:25230131-25459788 (GRCh38/Hg38). However, we obtained a PCR product of ~1800 bp (later determined to be 1832 bp by DNA sequencing) in all three affected individuals (Supplementary Fig. 4), suggesting that the genomic deletion eliminates nearly 228 kb DNA in members of PKCC212 harboring this deletion.

Sanger sequencing of the 1832 bp amplified PCR fragment identified 353 bp of *CRYBB2* and 1145 of *CRYBB2P1*, whereas the 334-bp sequence was indistinguishable due to the high sequence similarity between *CRYBB2* and *CRYBB2P1* (Fig. 2 and Supplementary Data 1). Moreover, we identified the c.463 C > T variant in exon 6 of *CRYBB2-CRYBB2P1*, which leads to a premature stop codon at glutamine 155 (p.Gln155*) in the PKCC212 family (Supplementary Fig. 5). All three affected individuals were homozygous for the variant, whereas unaffected individuals were either heterozygous or homozygous for the wild-type allele (Fig. 1a). This deletion was not present in 96 unaffected individuals of Pakistani descent who were examined to rule out the presence of cataracts and 24 unaffected individuals of Saudi descent.

Taken together, the abovementioned data suggest that intron 5 of *CRYBB2* became fused with intron 5 of *CRYBB2P1*, resulting in a hybrid *CRYBB2*-*CRYBB2P1* gene consisting of the first five exons and a part of intron 5 of *CRYBB2* followed by the remainder of intron 5, exon 6, and the 3’ UTR of *CRYBB2P1* (Fig. 2).

**Fig. 2:**
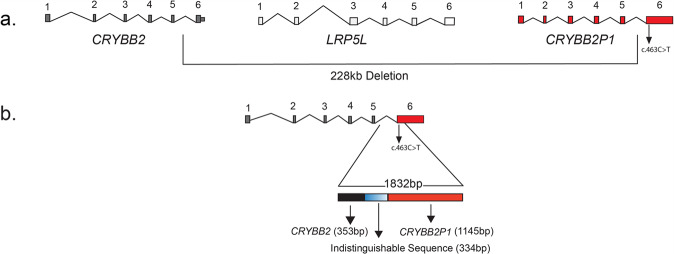
Schematic illustration of chromosome 22q genomic deletion in PKCC212. **a** A 228-kb genomic deletion of chromosome 22q was identified in PKCC212. **b** The deletion in affected individuals of PKCC212 removes exon 6 of *CRYBB2* (ENST00000651629.1), *LRP5 L* (ENST00000402859.6), and the first five exons of the *CRYBB2* pseudogene (*CRYBB2P1*). Amplification results using the primer pair BB2Int5_BB2P1Ex6 (Supplementary Table 2) identified a 1832-bp fragment of chromosome 22q. Of this 1832-bp region, 353 and 1145 bp aligned with *CRYBB2* and *CRYBB2P1*, respectively; 334 bp remained indistinguishable due to overwhelming similarity between *CRYBB2* and *CRYBB2P1*. Note: The *CRYBB2P1* transcripts, i.e., NR_ 033733.1 and NR_ 033734.1, consist of 5 and 6 exons, respectively. Exons 4 and 5 in the *CRYBB2P1* transcripts NR_ 033733.1 and NR_ 033734.1 are homologous to *CRYBB2* exon 6 harboring the p.Gln155* mutation. Note: Transcripts are as per Genome Reference Consortium Human Build 38 patch release 12 (GRCh38.p12) assembly.

We next investigated the whole exomes of the three affected individuals, i.e., individuals V:2, V:3, and V:4 of PKCC212, through next-generation sequencing. Whole-exome library preparation and next-generation sequencing of the affected individuals were performed commercially by Novogene Corporation Inc. (Davis, USA). The quality control analysis of exome data revealed >99% of the reads to be 150 base pairs, with 95% of the sequencing data yielding a PHRED score of 30 or above. High-throughput sequencing resulted in 48–66 million paired-end reads for each sample, and ~48–66 million reads (>99.8% of total reads) were uniquely mapped to the human genome (GRCh38.p13), representing an average 112× to 152× coverage for all three exomes (Supplementary Table 3). Lasergene Genomics Suite (DNASTAR, Madison, WI, USA) was used for reference-guided genome alignment and variant calling/annotation of the exome data. Paired-end raw reads were aligned to the human genome (GRCh38.p13), and the mapped reads were further processed for variant calling and annotation using DNASTAR (Madison, WI, USA) proprietary software (SeqMan NGen & ArrayStar Ver. 12) with default parameters. The stringent criterion was used to filter false-positive results from the potentially causal variants, as described^3^.

We first examined the linkage interval on chromosome 22q in the exome dataset, in particular, the 228-kb deletion. All variants (sequencing depth ≥5 reads) in the linkage interval were examined; however, we did not identify any variant originating from exon 6 of *CRYBB2* or *LRP5L* in the affected individuals (Supplementary Data 2, 3, and 4). To rule out the possibility of a causative variant other than chromosome 22q deletion either within the linkage interval or elsewhere in the exome being responsible for the cataractous phenotype in PKCC212, all variants present in the three exomes were systematically filtered through an approach previously adopted to identify mutation in *PEX5*^3^. Nevertheless, no variants in the exomes of individuals V:2, V:3, and V:4 justified the criterion for cataractogenesis (Supplementary Fig. 6).

Gene conversion resulting in the transfer of the c.463 C > T (p.Gln155*) variant from the pseudogene *CRYBB2P1* to the sixth exon of *CRYBB2* has been reported in multiple families with cataracts and manifests as an autosomal dominant trait^4–8^. In contrast, our data implicate a large genomic deletion encompassing exon 6 of *CRYBB2*, *LRP5L*, and most *CRYBB2P1* responsible for arCC in PKCC212. The precise pathogenic mechanism of cataractogenesis due to the fusion of *CRYBB2* and *CRYBB2P1* remains unclear. It is noteworthy that the 3’UTR of hybrid *CRYBB2* originates from the pseudogene that is not transcribed and/or translated and therefore most likely lacks the ability to protect nascent mRNA against degradation^9^. Given the importance of the 3’UTR in protecting mRNAs from degradation, it is tempting to speculate that the mRNA of the hybrid *CRYBB2* (which includes the c.463 C > T (p.Gln155*) mutation) is not stable and is degraded. This would result in only wild-type mRNA being present in heterozygous carriers. We further speculate that a single functional *CRYBB2* allele in heterozygous carriers is adequate for lens transparency. Conversely, individuals homozygous for the mutant allele completely lack functional CRYBB2, leading to a null phenotype and congenital cataracts.

Recently, Sun et al. reported a novel pathogenic mutation in *LRP5L* (c.107 C > G, p.P36R) in a congenital membranous cataract family^10^. Despite the possibility that concurrent deletion of *LRP5L* contributed to cataractogenesis in PKCC212, it is unlikely given the presence of null alleles in gnomAD database (https://gnomad.broadinstitute.org).

In conclusion, we identified a homozygous genomic deletion resulting in a hybrid *CRYBB2* responsible for arCC. To the best of our knowledge, this is the first study reporting a large genomic deletion and subsequent fusion of *CRYBB2-CRYBB2P1* being responsible for arCC. Mutations in all three β-crystallin genes, *CRYBB1*, *CRYBB2*, and *CRYBB3*, have been associated with arCC^11,12^, suggesting that haploinsufficiency of β-crystallins is tolerated by the ocular lens without affecting their respective physiological function in the lens.

## Supplementary information

Supplementary Material

Supplementary Data 1

Supplementary Data 2

Supplementary Data 3

Supplementary Data 4

## Data Availability

The relevant data from this Data Report are hosted at the Human Genome Variation Database at 10.6084/m9.figshare.hgv.3216.
