# Supplementary material for: A genomic deletion encompassing CRYBB2-CRYBB2P1 is responsible for autosomal recessive congenital cataracts
Source: Hum Genome Var. 2022 Sep 8;9:31. doi: 10.1038/s41439-022-00208-7 (PMC9458725; doi:10.1038/s41439-022-00208-7)
Supplement: Supplementary file 1 — Supplementary Material [file 41439_2022_208_MOESM1_ESM.pdf]

A genomic deletion encompassing *CRYBB2-CRYBB2P1* is responsible for autosomal recessive congenital cataracts

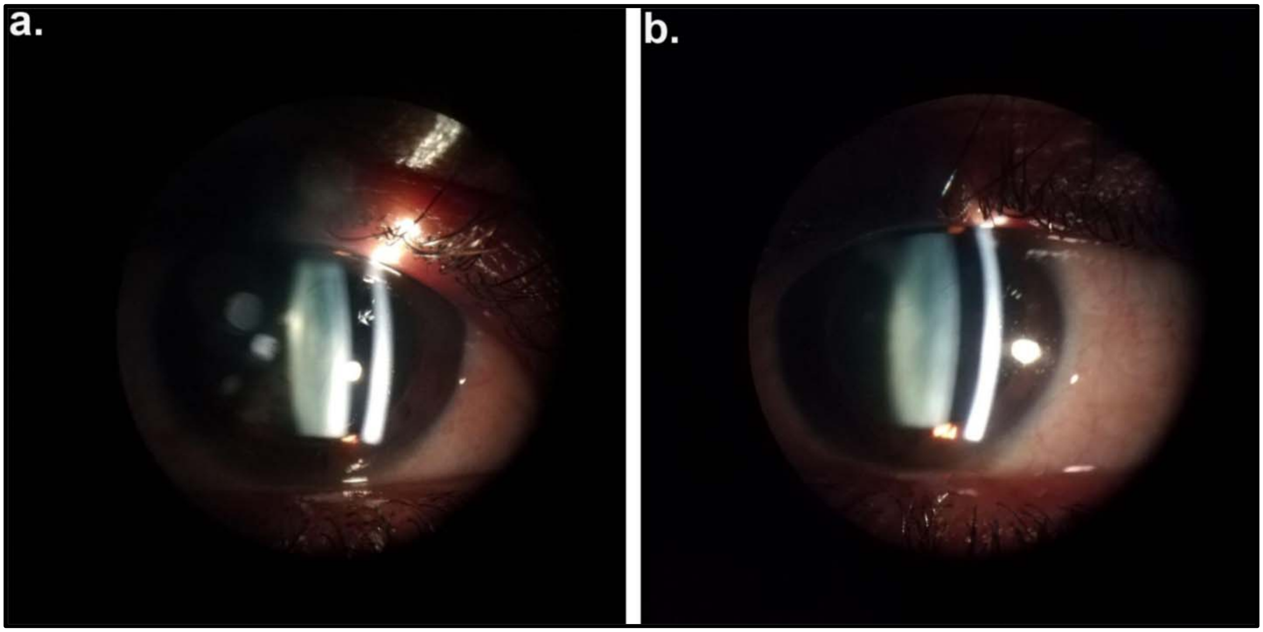

Supplementary Figure 1: Slit-lamp photographs of the unaffected individual IV:3 of PKCC212 (age, 41 years), show a clear lens with no cataracts. **a)** Oculus dexter (right eye); **b)** Oculus sinister (left eye). **Note:** Haplotype analysis coupled with PCR amplifications determined that Individual IV:3 is heterozygous for chromosome 22q genomic deletion.

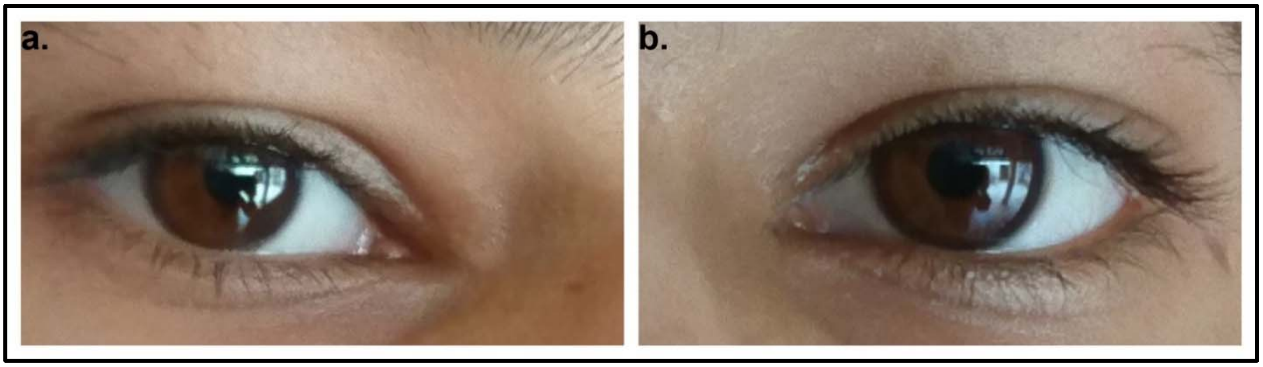

Supplementary Figure 2: Anterior front view of the eyes of individual V:1 of PKCC212 (age, 16 years), the older sibling of the three affected individuals show a clear lens with no cataracts. **a)** Oculus dexter (right eye); **b)** Oculus sinister (left eye). **Note:** Haplotype analysis coupled with PCR amplifications determined that Individual V:1 is heterozygous for chromosome 22q genomic deletion.

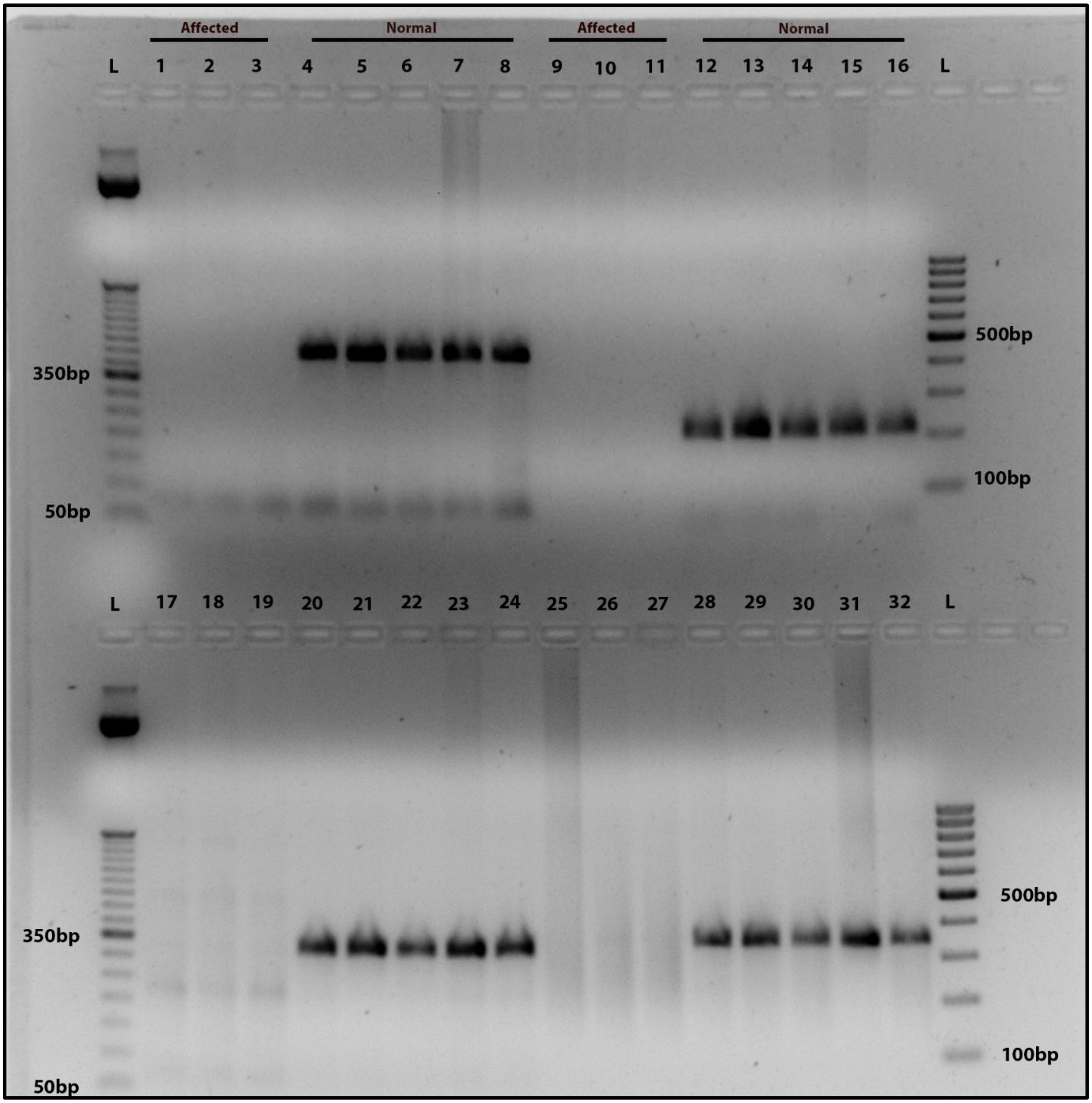

Supplementary Figure 3: Agarose gel electrophoresis of PCR products of affected and unaffected members of PKCC212 obtained from four primers amplifying different regions between homologous genes; *CRYBB2, CRYBB2P1*. Lane L: DNA ladder,1-8: PCR amplification of primer BB2_LRP, 9-16: amplification of primer LRP5L_Ex2, 17-24: amplification of primer LRP5L_Int3, and 25-32: amplification of primer BB2P1_Int. **Note:** affected individuals (V:2, V:3 and V:4) in lanes: 1-3, 9-11, 17-19, 25-27 respectively and unaffected individuals (III:3, IV:2, IV:3, V:1 and V:5) in lanes: 4-8, 12-16, 20-24, 28-32 respectively.

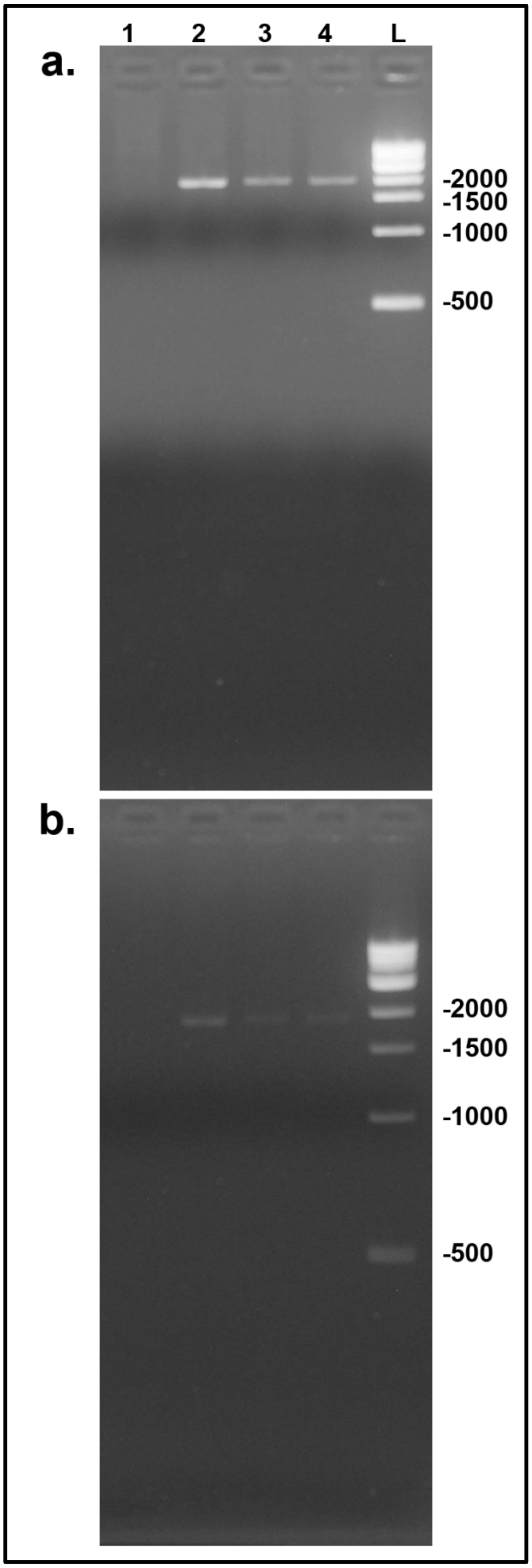

Supplementary Figure 4: Agarose gel electrophoresis of PCR amplicons obtained by the forward primer annealing to *CRYBB2* and a reverse primer annealing to *CRYBB2P1*. PCR amplicons of the forward primer (BB2Int5) and reverse primer (BB2P1Ex6), located in *CRYBB2* and *CRYBB2P1*, respectively, which theoretically would amplify a 230 kb region. **a)** 30 minutes electrophoresis; **b)** 90 minutes electrophoresis. No PCR amplification in lane 1 (genomic DNA of an unaffected, unrelated control) while PCR fragment of nearly 1800 is present in lanes 2-4 (genomic DNAs of affected individuals V:2, V:3, and V:4 respectively). **Note:** lane L: DNA ladder.

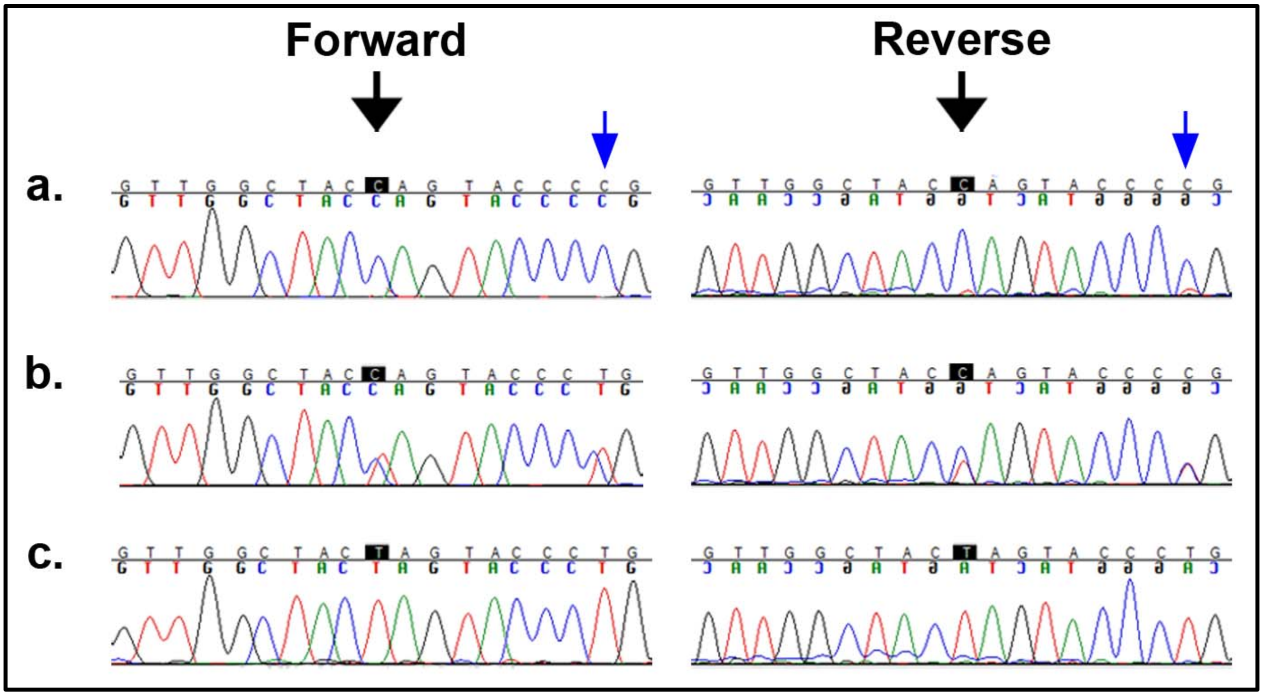

Supplementary Figure 5: Sanger sequencing of the amplicons in PKCC212 generated by PCR using forward (CTCGCCTCTCTCTCTGTCTG) and reverse (AGCAGACAAGTTGCAAGTCA) primers. Sequence chromatograms of **a)** individual V:5 (unaffected), homozygous for the wild type allele; **b)** individual V:1 (unaffected) heterozygous and **c)** individual V:3 (affected), homozygous carrier of the c.463C>T variation. **Note:** The arrows in black point to the c.463C>T variant while, the blue arrows indicate another transition c. 471C>T that does not alter the amino acid sequence.

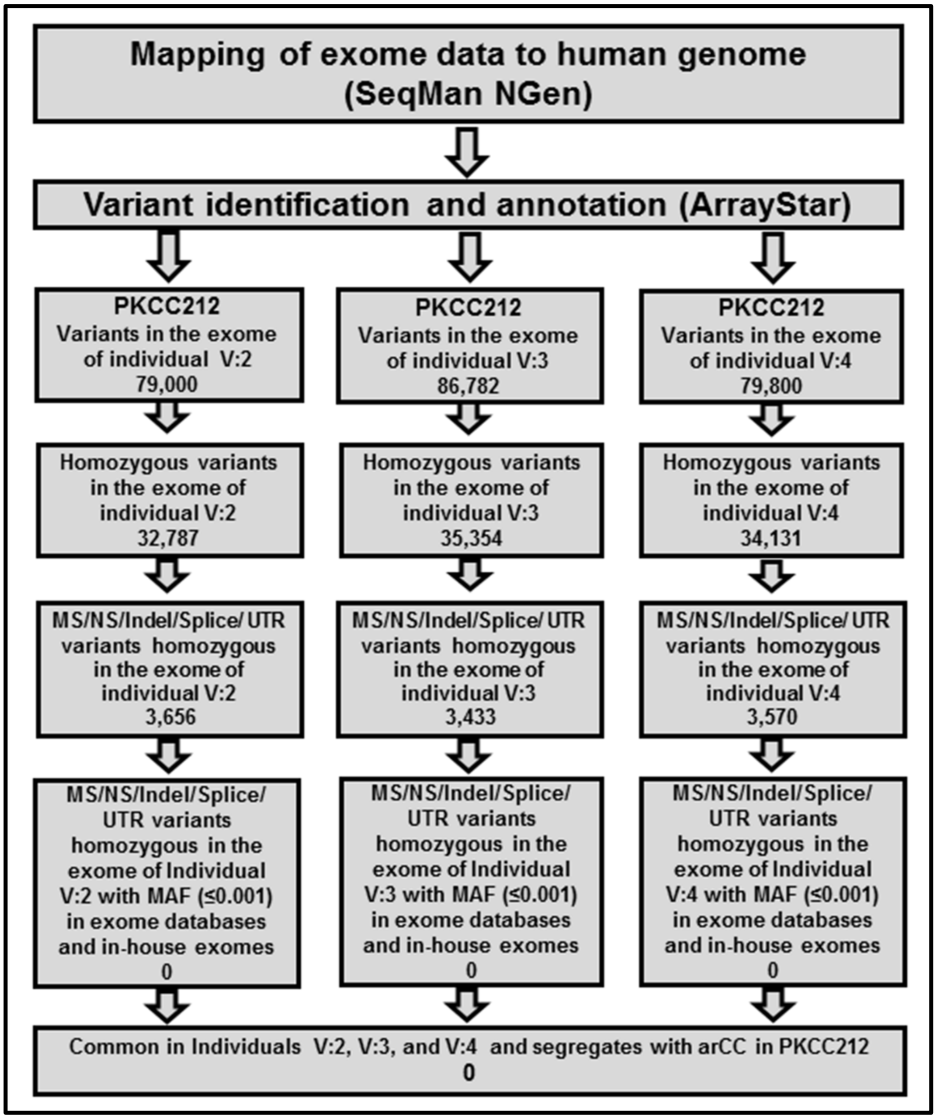

Supplementary Figure 6: Flow chart depicting the protocol used for the bioinformatic analysis of whole-exome sequencing data. The paired-end reads were aligned to the human genome (GRCh38.p13) using SeqMan NGen (Ver. 12; DNASTAR) and mapped reads were processed for variant calling and annotation with ArrayStar (Ver. 12; DNASTAR). The non-synonymous homozygous variants in the coding regions of the genome segregating in affected individuals V:2, V:3, and V:4 of PKCC212 were examined. The variants with a minor allele frequency (MAF) greater than 0.001% in public databases and in-house exome datasets were excluded from the analysis. **Note:** MS; Missense, NS; Nonsense, Indel; Insertion/deletion, UTR; untranslated region, MAF; Minor allele frequency, and N.A.; Not applicable.

Supplementary Table 1: Two-point LOD scores of PKCC212 for alleles of chromosome 22q11.23 microsatellite markers.

Markers	cM	Mb	0	0.01	0.03	0.05	0.07	0.09	0.1	0.2	0.3	Z_{max}	θ_{max}
D22S427	8.32	18.1	1.98	1.93	1.84	1.74	1.64	1.55	1.50	1.00	0.51	1.98	0.00
D22S539	14.44	21.9	1.98	1.93	1.84	1.74	1.64	1.55	1.50	1.00	0.51	1.98	0.00
D22S686	13.6	22.72	2.28	2.23	2.12	2.02	1.92	1.81	1.76	1.24	0.73	2.28	0.00
D22S1174	19.32	24.09	2.21	2.16	2.07	1.97	1.88	1.78	1.73	1.23	0.74	2.21	0.00
D22S315	21.47	25.61	2.51	2.46	2.36	2.25	2.15	2.05	1.99	1.47	0.95	2.51	0.00
D22S1154	23.37	26.22	2.28	2.23	2.14	2.04	1.94	1.85	1.80	1.29	0.79	2.28	0.00
D22S689	28.02	28.46	$-\infty$	-1.53	-0.66	-0.30	-0.10	0.04	0.09	0.27	0.22	0.27	0.20

Supplementary Table 2: The primer sequences and amplification conditions.

Gene	Exon	Genomic Coordinates (GRCh38/hg38, 2013)	Forward Primer	Reverse Primer	Annealing Temp (°C)
CRYBB1	2	chr22: 26616029-26616444	TAGAGAGGAAACGAGCTCCAAG	AGGTGCGGAGGAGTAAGAGG	68-58
	3	chr22: 26611999-26612279	GCACTGCTGGCTTTTATTTATG	AGAAATGGCAGCTACTGTTGTG	68-58
	4	chr22: 26607784-26608106	AGGGGAGAGAGAAAGGCAAG	CTCCCTACCCACCATCATCTC	68-58
	5	chr22: 26601818-26602088	CCCGCTAAGTTTCTTCTCTTTG	AGCCTCTGATTCTGCCTGTG	68-58
	6	chr22: 26599328-26599768	AGGGATCAATGAAGGACAGG	GGAAGTCACATCCCAGTAACTATG	68-58
CRYBB3	2	chr22: 25201316-25201530	TCACATCAACACCTGGCTTC	AAGATGACCCTGAGGCCC	68-58
	3	chr22: 25202595-25202851	CTCTAATGCCCAAAGGAGGG	CCTCCACACTCCCAGAAGG	68-58
	4	chr22: 25203702-25203950	AAACTTGAATCCTTCCTCAGC	TCTCAGTGCACCCTGCTTC	68-58
	5	chr22: 25205154-25205431	CTGGAGCCTCCTTGACCTC	TGATTCTGTTTGGAGCCAGAG	68-58
	6	chr22: 25206992-25207275	GAGGCAGGGTGACTGGAAG	CCTCCTCTTCTTGCCCTTG	68-58
CRYBA4	2	chr22: 26622451-26622705	AGCCATGCATTGCCCCTA	TCCTAGGATTCATGGGGACCT	68-58
	3	chr22: 26623126-26623452	GAGTTTGCAATCCCTGCTTT	CTAGGGAGAGGGGACCTAGAA	68-58
	4	chr22: 26625400-26625682	CCGTTCTAGACCCAATTGCTG	TCCGAAGTGCCCACATGA	68-58
	5	chr22: 26628231-26628490	GCTCCTGGGTTTCCAACTG	GGTACACCTACCCCTCCCAGTA	68-58
	6	chr22: 26630263-26630626	ATAGATCCCTTTGCCCCTGT	ACTGTGCACGGACCAGTTC	68-58
CRYBB2	2	chr22: 25221332-25221560	TCTCAAGGCCCCACAGAGT	GCCAAGCCCATTTTACAGAA	68-58
	3	chr22: 25224747-25225133	ACGGCTGCTTATAGCCAGAG	CAGATTTGCAGACAGGAGCA	68-58
	4	chr22: 25227687-25228032	GGGATTTTGCACTTGGATTT	AGGATGATGGGCAGAGAGAG	68-58
	5	chr22: 25229304-25229632	GTAGTGGGTGCACTGGGAAG	CCCCAGAGTCTCAGTTCCTG	68-58
	6	chr22: 25231315-25231975	CTGACCCCAGTACAGTACAGT	CATTTCTCTCTCGCTGTCACTCTCTC	68-58
**Additional Primers**	CRYBB2_Int	chr22: 25230138-25230840	TACCAGCTTGTAAGAACTTTTT	TCATGTGAATTCTTAAAGTATTT	68-58
	CRYBB2K1	chr22: 25232039-25232429	GCAGCTGCTTAGCTGTGTG	AGAGGTTGCGTAGATTCTTCAA	68-58
	CRYBB2K2	chr22: 25233048-25233707	AGACGCAGTTTCACCACGTT	GCAGTGGTCCCCAAATGTT	68-58
	CRYBB2K3	chr22: 25234033-25234860	GGACATAGGGAGGGGAACAT	CTTTGGCCTCTTTACCCTTG	68-58
	CRYBB2K4	chr22: 25235123-25235716	TGGGCAGAGTAGGGATACAAA	CATAGACACCAGTCACATTCCA	68-58
	BB2_LRP	chr22: 25284589-25285021	CCAGCAAATGGGAAACTTAGAGA	ACAGGCATGAGCCACTATCAC	68-58
	LRP5L_Ex2	chr22: 25357261-25357467	AGACAGATGCATGGGGTCAT	CTTGTCTGTCTTGGCATCTCC	68-58
	LRP5L_Int3	chr22: 25352495-25352808	CCATGGTTTACTAAAGGAGATGAAC	CTCCGACCCAGGAAGAACA	68-58
	BB2P1_Int	chr22: 25454736-25455077	AGAGTTTCACTGGTCTCGAAC	GGCATGGCTAATAGAAGAAAGC	60-50
	BB2Int5_BB2P1Ex6	chr22: 25230131-25459788	CAGCTTATACCAGCTTGTAAGAACTTT	TAGCCCAGATACTTGCCAATTC	68-58

Supplementary Table 3: Statistics of next-generation based whole-exome sequencing data.

Individual ID	Total Reads (10^6)	Mapped Reads (10^6)	% Mapped Reads	Sequenced Bases (Mb)	Exome coverage (x)
V:2	48.83	48.77	99.86	7315.80	112.55
V:3	66.25	66.19	99.91	9929.84	152.76
V:4	60.90	60.84	99.91	9127.41	140.42

Supplementary Data 1: Sanger sequencing shows that the intron 5 of *CRYBB2* fused with the intron 5 *CRYBB2P1* resulting in a hybrid *CRYBB2-CRYBB2P1* gene consisting of the first five exons and a part of intron 5 from *CRYBB2* followed by the remainder of intron 5 and exon 6 from *CRYBB2P1*. Sanger sequencing confirmed that the *CRYBB2* hybrid contains the exon 6 and 3' UTR of *CRYBB2P1*.

Supplementary Data 2: The list of variants identified in the affected individual V:2 of PKCC212 by next-generation based whole-exome sequencing.

Supplementary Data 3: The list of variants identified in the affected individual V:3 of PKCC212 by next-generation based whole-exome sequencing.

Supplementary Data 4: The list of variants identified in the affected individual V:4 of PKCC212 by next-generation based whole-exome sequencing.
