# Supplementary material for: A genomic deletion encompassing CRYBB2-CRYBB2P1 is responsible for autosomal recessive congenital cataracts
Source: Hum Genome Var. 2022 Sep 8;9:31. doi: 10.1038/s41439-022-00208-7 (PMC9458725; doi:10.1038/s41439-022-00208-7)
Supplement: Supplementary file 2 — Supplementary Data 1 [file 41439_2022_208_MOESM2_ESM.pdf]

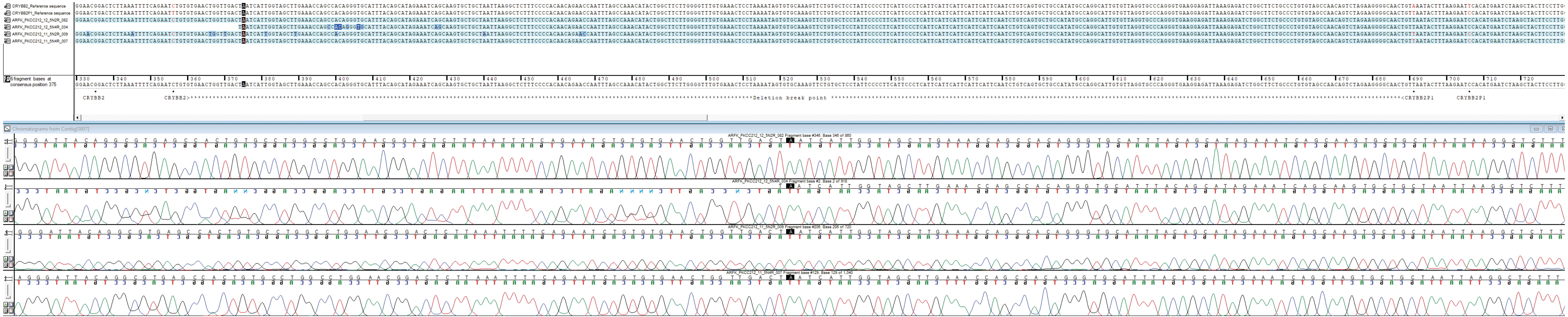

CRYBB2_Reference sequence
CRYBB2P1_Reference sequence
ARFK_PKCC212_12_5N2R_082
ARFK_PKCC212_12_5N4R_034
ARFK_PKCC212_11_5N2R_009
ARFK_PKCC212_11_5N4R_007
5 fragment bases at consensus position 375
CRYBB2
CRYBB2>
Deletion break point
<CRYBB2P1
CRYBB2P1
Chromatograms from Contig[0007]
